# tRNA Modifications and Modifying Enzymes in Disease, the Potential Therapeutic Targets

**DOI:** 10.7150/ijbs.80233

**Published:** 2023-02-13

**Authors:** Weifang Cui, Deze Zhao, Junjie Jiang, Faqing Tang, Chunfang Zhang, Chaojun Duan

**Affiliations:** 1 Department of Thoracic Surgery, Xiangya Hospital, Central South University, Xiangya Road 87th, Changsha, 410008, Hunan, PR China.; 2 Hunan Engineering Research Center for Pulmonary Nodules Precise Diagnosis & Treatment, Changsha, 410008, Hunan, PR China.; 3 National Clinical Research Center for Geriatric Disorders, Changsha, 410008, Hunan, PR China.; 4 Institute of Medical Sciences, Xiangya Lung Cancer Center, Xiangya Hospital, Central South University, Changsha 410008, Hunan, PR China.; 5 Hunan Key Laboratory of Oncotarget Gene, Hunan Cancer Hospital & The Affiliated Cancer Hospital of Xiangya School of Medicine, Central South University, Changsha 410008, Hunan, PR China.

**Keywords:** tRNA modification, tRNA modifying enzyme, signaling pathways, biomarkers

## Abstract

tRNA is one of the most conserved and abundant RNA species, which plays a key role during protein translation. tRNA molecules are post-transcriptionally modified by tRNA modifying enzymes. Since high-throughput sequencing technology has developed rapidly, tRNA modification types have been discovered in many research fields. In tRNA, numerous types of tRNA modifications and modifying enzymes have been implicated in biological functions and human diseases. In our review, we talk about the relevant biological functions of tRNA modifications, including tRNA stability, protein translation, cell cycle, oxidative stress, and immunity. We also explore how tRNA modifications contribute to the progression of human diseases. Based on previous studies, we discuss some emerging techniques for assessing tRNA modifications to aid in discovering different types of tRNA modifications.

## Introduction

**Figure 1:**
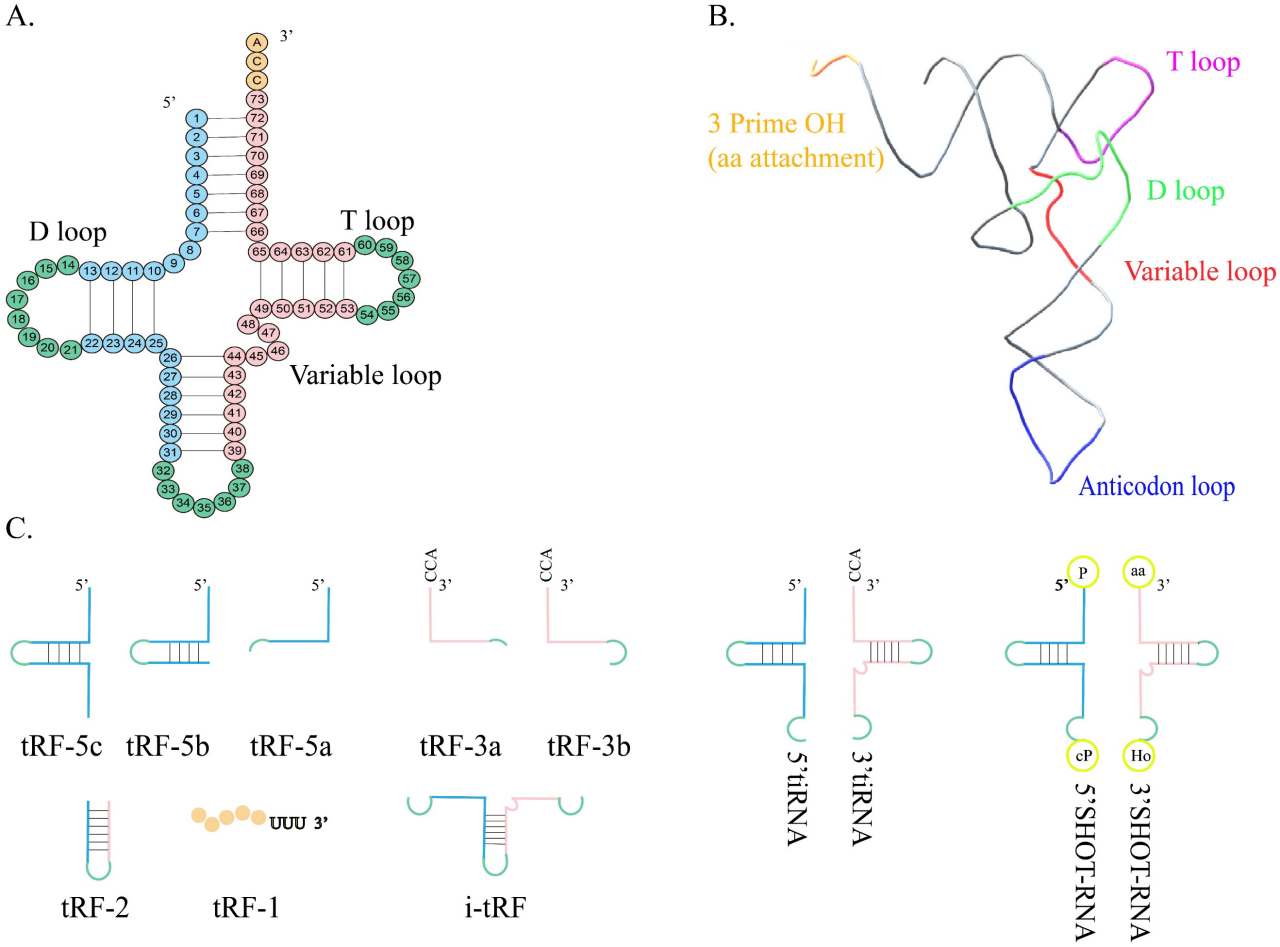
** Structure and classification of tRNA. A.** The shamrock-like secondary structure of tRNA. **B.** The L-shaped tertiary structure of tRNA. **C.** tRF-5 is formed by the Dicer cleaving in the D loop or between the D loop and the anticodon loop of mature tRNA. tRF-3 is the 3' terminal portion of mature tRNA, containing the CCA sequence. tRF-2 contains anticodon stem-loop sequences but lacks the 5' end and 3' end of tRNA. tRF-1is derived from the cleavage of the 3' terminal of the pre-tRNA. i-tRF is generated from the interior of mature tRNA spanning anticodon regions. 5'tiRNA is from the 5' terminal of the mature tRNA to the end of the anticodon loop, and 3'-tiRNA is from the 3' end to the end of the anticodon loop. SHOT-RNA is produced in response to sex hormone stimulation and engaged by ANG.

With the rapid development of high-throughput sequencing technology, tRNA-related studies are becoming more common. The classical role of tRNA is to synthesize proteins from different amino acids in the ribosome via the guidance of mRNA. In the process of biogenesis, tRNA is cleaved into different small fragments by specific enzymes including angiogenin (ANG), Dicer, and other RNA enzymes^1^, ^2^. These fragments are called tRNA-derived small RNA fragments (tsRNA) and can generally be divided into the following two subtypes: tRNA-half, also known as tRNA-derived stress-induced RNA (tiRNA), and tRNA-derived fragments (tRFs) (Figure 1). tsRNA is very abundant and has been identified in all three life domains, including archaea, bacteria, and certain unicellular organisms^3^, ^4^. tsRNA is not a randomly degraded tRNA fragment, but its production is related to tRNA chemical modification and has a precise sequence and rich biological functions^5^-^8^. At present, some public databases can continuously identify new tsRNA and predict their downstream targets, which helps the understanding of tRNA^9^.

So far, more than 200 RNA modifications are known in all areas of life^10^, about half of which are present in tRNAs, including methylation, acetylationdeamination, isomerization, glycosylation, thiolation reactions and pseudouridylation^11^, and their frequency and distribution depend on the organism or tRNA species^12^.

Among the modifications, methylation of tRNAs is one of the most prominent post-transcriptional modifications, occurring on nitrogen rings of almost all bases^13^, mainly including N-methyladenosine/guanosine, N-methylcytidine, and 5-Methyluridine (m^5^U). tRNA methylation is crucial for its maturation and function execution. Methylation of tRNAs is catalyzed by methyltransferase, including the TRM10, NSUN families, and METTL families, with S-adenosylmethionine as the methyl donor, While ALKB family members catalyze demethylation^14^, ^15^.

N^4^-acetylcytidine (ac^4^C) is usually thought of as a conservative chemically modified nucleoside present on tRNAs and rRNAs. According to studies, ac^4^C can only exist at position 12 in eukaryotic tRNA^16^, ^17^. The ac^4^C of tRNA is produced by *N*-acetyltransferase 10 with the help of THUMPD1, which is combined with tRNA^18^.

Pseudouridine (Ψ) is by far the most abundant type of tRNA modification known. In eukaryotes, Ψ synthases are known as PUS, divided into 10 different types, called PUS1 to PUS10^19^. They modify uridine into Ψ by recognizing the secondary structural elements and sequence of the target uridine-containing RNA.

tRNA is a nice model for researching RNA modifications, this is because of the high cellular abundance of tRNA and the existence of a mass of modifications on tRNA. Base modifications can affect many aspects of tRNA functions, such as ensuring correct folding and stable tertiary structure of tRNA, ensuring accurate and efficient translation^20^, and affecting cell heat resistance and stress^21^. Due to the importance of tRNA modifying enzymes in protein synthesis, a large number of human diseases are shown to be related to aberrant expression and dysfunction of tRNA modifying enzymes^22^, including neurological disorders^23^, mitochondrial diseases^24^, ^25^, cancer^26^ and diabetes mellitus^27^. Therefore, a comprehensive understanding of tRNA modifications is so necessary for us that we can better understand the physiological and pathological processes of tRNA-mediated human diseases.

Studying the biological function of tRNA modifications relies on continuous advances in technology to detect modifications. However, due to the complexity and specificity of RNA modifications, the detection, localization, and quantification of RNA modifications have always been a technical challenge, which has resulted in limited research on tRNA modifications in the past^28^. New technologies for RNA modification detection are constantly evolving, opening the door to a new world of tRNA modifications, but there are still some unavoidable limitations^29^.

**Figure 2:**
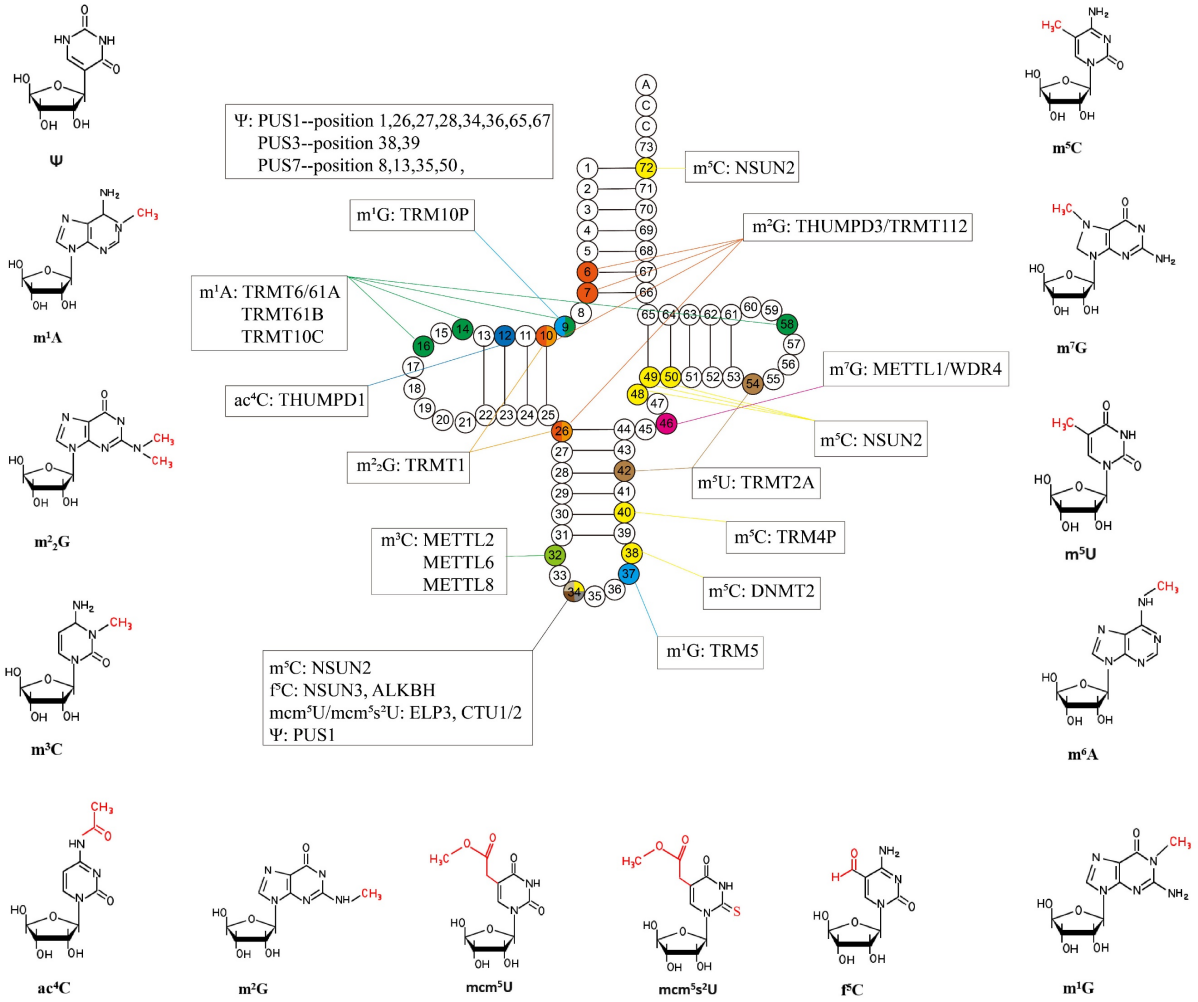
** Common tRNA modifications and their chemical structures discussed in this paper.** Ψ: Pseudouridine; m^1^A: N1-methyladenosine; m^2^G: N2, N2-dimethylguanosine; m^5^C: 5-methylcytodine; ac^4^C: N4-acetylcytidine; m^2^G: N2-methylguanosine; mcm^5^U: 5-methoxycarbonylmethyluridine; mcm^5^s^2^U: 5- methoxycarbonylmethyl-2-thiouridine; f^5^C: 5-formylcytidine; m^3^C: 3-methylcytodine; m^7^G: N7- methylguanosine; m^1^G: N1-methylguanosine; m^6^A: N6-methyladenosine; m^5^U: 5-methyluridine.

In this review, we focus on the relationship between tRNA modifications and tRNA structural stability, protein translation, cell cycle, oxidative stress, and immunity. In addition, we summarize the common types of tRNA modifications (Figure 2) and assess their possibility as novel diagnostic markers and clinical therapeutic targets in human diseases. Finally, we discuss the current limitations of this field and look ahead to where it was going in the future.

## tRNA modifications and Biological processes

### tRNA modifications and tRNA structural stability

The disruption of Watson-crick base pairing consequently affects the structure of RNAs and thereby influences gene expression and their functions^30^. Similarly, tRNA modifications can affect the secondary and tertiary structure of tRNA by disrupting Watson-crick base pairing. In tRNAs, most of the base modification sites are concentrated in two regions. One is located at the core region of the tertiary structure of tRNA and the other is in the anticodon domain^11^. The effect of modifications on the structure relies on their type and location in the tRNA, including the effects on the hydrophobic character of a base, base pairing and stacking, and charge stabilization of nucleotides^21^, ^31^.

Modifications that occur at the core region of the tRNA structure, that is in the D-loop or T-loop, are necessary for tRNAs to maintain secondary and tertiary structural stability, particularly methylation modifications^31^. The m^1^A modifications occurring at site 9 (m^1^A_9_) of human mt-tRNA^Lys^ damage the formation of the Watson-Crick base pair within the stem. Similar to m^1^A, m^1^G disrupts canonical base pairing by forming methyl groups on tRNA bases and thus blocking the formation of the Watson-Crick face, thereby interfering with secondary structure formation^32^. In addition, tRNA modifications can stabilize the structure of tRNAs by providing a hydrophobic or hydrophilic environment for the bases of the local structures. The m^5^C_48_ strengthens the hydrophobicity between the bases, and contributes to the stacking of bases, thus stabilizing the L-shaped tertiary structure of the tRNAs^33^. The m^5^C_40_ promotes the binding of Mg^2+^ to tRNA^34^, which is known to stabilize the tertiary structure of tRNAs^35^. By disrupting Watson-Crick base pairing and stabilizing the core of L-shaped structure, m^2^G_10_ and m^2^G_26_ promote the secondary and tertiary folding of tRNAs^31^.

In summary, methylation modifications that occur on tRNAs generally deny Watson-Crick and canonical base pairing. Furthermore, the side chain of N6-threonylcarbamoyladenosine (t^6^A) by intramolecular hydrogen bonds to make its planar ring be extended, by enhancing π-π stacking with neighboring bases and preventing pairing with the base at position 33 to stabilize the anticodon loop structure^36^. Ψ is widely distributed on the tRNA structure and also helps maintain the correct shape of the tRNA, which is related to its increased hydrophobicity on the one hand, and H bond formation in the anticodon ring in the tRNA on the other hand^19^, ^37^.

Positive charges at the TΨC domain and the D-loop are related to the tertiary folding of tRNAs^38^. There is evidence that both m^7^G and m^1^A can influence the formation of non-Watson/Crick hydrogen bonds by carrying positive charges, thereby affecting the stability of tRNA structures^39^. In mitochondrial tRNA, the m^3^C_32_ modifications are associated with the folding of mt-tRNA^Ser/Thr(UCN)^, this is because the m^3^C_32_ brings in a positive charge to strengthen electrostatic stability^40^.

Furthermore, hypomodified tRNAs become more sensitive to the RNA degradosome and prone to rapid degradation^41^. Therefore, modified nucleotides affect the folding and structure of tRNA in a variety of ways, which is conducive to the construction of the most efficient ASL conformation for effective translation. It is worth noting that although some modifications do not affect the overall structure, they can modulate local dynamics, make tRNA's secondary and tertiary structure harmonized^42^.

### tRNA modifications at anticodon and Protein translation

Most post-transcriptional modifications occur at the anticodon loop of tRNA, which is crucial for the accurate synthesis of proteins at different translation steps, such as aminoacylation, decoding and translocation^43^, especially at positions 34 and 37.

According to the classical wobble hypothesis in 1966, position 34 at the first anticodon is known as the “wobble” position^44^. Nucleosides at this site can bind to non-standard bases, which is called the degeneracy of codons. The modified wobble hypothesis was proposed in 1991, suggesting that specific base modifications select particular codons^45^. Since then, increasing evidence has shown that nucleoside modifications at tRNA positions 34 and 37 are crucial for the accurate and effective translation of genetic code, and modification at position 34 can limit or extend the decoding ability of tRNA^46^-^48^. The low modification state of the 34th position is not conducive to the combination of codons and anticodons and affects the fidelity of translation^49^. In addition, modification of position 34 was found to directly affect the stability of the anticodon ring plane base and thus affect translation^50^.

Hypomodified tRNAs, such as lack of mcm^5^s^2^U_34_, cannot efficiently decode their cognate codons, resulting in ribosome suspension, thereby impinging protein homeostasis^51^. Q_34_, which occurs on mitochondrial tRNA, is thought to promote tyrosine translation in mitochondria^52^. The ac^4^C at the tRNA^Met^ wobble position can improve the accuracy of tRNAs reading noninitiating AUG codons and weaken the affinity between tRNA and the codon AUG^53^, as a result, the translation of the codon is reduced and ultimately affect protein synthesis. Moreover, the "distal" conformation of ac^4^C can hinder the misreading of the AUA codon during protein translation^54^.

In addition, I_34_, f^5^C, and m^5^C also extend the decoding capability of tRNA^55^. In tRNA, m^5^C has been demonstrated to optimize codon-anticodon pairing and control translation efficiency and accuracy^56^. The m^7^G for the corresponding tRNAs might affect ribosome translocation. The lack of METTL1 promotes the movement of ribosomes on mRNAs, resulting in a higher frequency of m^7^G-tRNA decoding, indicating that m^7^G-tRNA modification and expression of related modifying enzymes affect translation efficiencies^57^-^59^.

The type of modification at tRNA position 37 depends on the base at position 36^60^, mainly to improve the stability of the codon and anti-codon coupling via cross-stacking with the first base of the codon^61^, ^62^. t^6^A is a generally conserved modification located at position 37 of tRNA and can promote tRNA binding to the A-site codon and efficient translocation, ensuring the efficiency and correctness of translation^36^.

Studies of pseudouridine synthetase (Pus1) in S. cerevisiae have shown that Pus1-dependent pseudouridylation is very important for particular decoding events *in vivo*. Pus1 deletion markedly increased the codon misreading of CGC (Arg) by tRNA^His^^63^.

These modifications collectively affect the binding of codons to anticodons and thus affect the protein translation process.

### tRNA modifications and Cell cycle

Modification of tRNA is related to the cell cycle. The level of tRNA modifications affected the aggregation status of Cdc13 and cell division^64^. It has been found that irreversible cell cycle arrests both in G1 and G2 in tad3-1 mutant cells because of an impairment in the A to I conversion at position 34 of tRNA^65^. Interestingly, there was an increase in the S phase, relative to G1 and G2, in Trm9 mutant cells due to the lack of Trm9-dependent tRNA modification (mcm⁵U)^66^. Further research has revealed that mcm⁵U affecting the cell cycle may have been achieved through the reciprocal regulation of TORC signaling and tRNA modifications by Elongator^67^. The percentage of G2 phase is increased when METTL1 knockout in mouse embryonic stem cells and intrahepatic cholangiocarcinoma (ICC) cell, lead to slower cell proliferation and colony formation ability is impaired, revealing the key role of METTL1-mediated m^7^G tRNA modification for the regulation of cell cycle^57^, ^58^. Besides, TRMT2A, which is involved in the 5-methylation of uracil located at position 54 (m^5^U_54_) of tRNA, is a potential regulator of the cell cycle in mammals^68^.

### tRNA modifications and Oxidative stress

**Figure 3:**
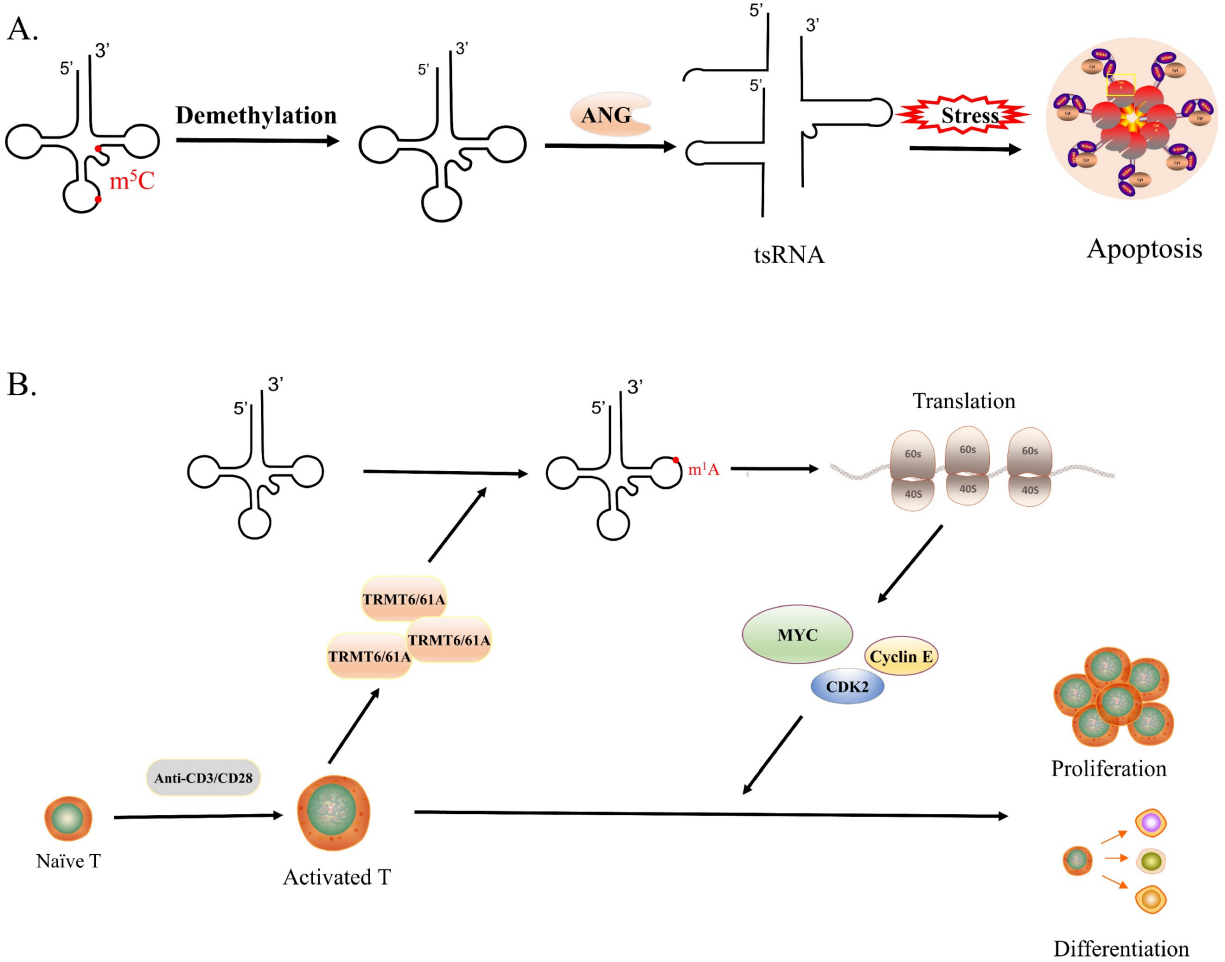
** tRNA modifications and biological processes. A.** The lack of m^5^C modification would increase the affinity between tRNA and ANG, leading to the production of large amounts of tsRNA, which would make cells more sensitive to oxidative stress and promote cell apoptosis. **B.** The expression of TRMT6/61 increases rapidly in the short term after T cell activation, which generates a large number of tRNA-m^1^A_58_, promoting translation and synthesizing a large number of functional proteins timely, in turn, driving the rapid proliferation and differentiation of activated T cells.

Quantitative mass spectrometry allows one to assess and compare changes in tRNA modifications abundance, which provides clear evidence that tRNA modifications, especially methylation, have a relationship with stress^69^. Specific m^5^C regulates the cellular stress response. Under oxidative stress, the amount of m^5^C at site 48 in tRNA decreased and the modification of m^5^C at the oscillating position increased^31^. The loss of NSUN3-mediated m^5^Cs in the anti-codon ring of mitochondrial tRNA^Met^ results in reduced induction of mitochondrial ROS under stress^70^. Deletion of NSUN2-mediated tRNA methylation makes cells more sensitive to oxidative stress stimulation, while NSUN2-overexpressing cells had higher cell viability under stress (Figure 3A)^71^. In addition, after heat shock DNMT2 relocalizes to stress particles, and stress-induced cleavage of tRNAs depends on DNMT2^72^. These observations suggest that tRNA methylation is related to oxidative stress in cells.

Other studies have suggested that some rare types of tRNA modifications are also involved in oxidative stress. Biochemical experiments revealed that 3-(3-amino-3-carboxypropyl) uridine U_47_ (acp^3^U_47_) confers thermal stability on tRNA. When tRNA aminocarboxypropyltransferase, which is in charge of the formation of acp^3^U in tRNA, is knocked out of the Escherichia coli, it causes genome instability in E. coli. under sustained heat stress^73^*.* It has been reported that genetic analysis showing MNMA, known as the enzyme that catalyzes sulfuration of uridine at position 34 in tRNAs, is important for sustaining oxidative stress^74^.

### tRNA modifications and Immunity

Certain tRNA modifications are associated with infection and regulate immune function^75^. With the development of new methods, the regulation of cellular immunity mediated by tRNA modifications will become the focus of research. Guanosine 2′-*O*-methylation located at position 18 (Gm_18_) in bacterial tRNA has been revealed to antagonize tRNA-induced TLR7/8 activation, indicating the modulation of 2′-O-methylations in tRNAs participates in immune escape^76^. Similarly, data from Adeline Galvanin et al further suggest that under starvation and antibiotic stress conditions, Gm_18_ methylation in tRNA selectively increases, which inhibits the host immune response by the RNA/TLR7 axis^77^. However, human tRNA^Lys^_3_ was immunosilent despite lacking Gm18, it is due to the 2′-O-methylthymidine modification at position 54 which limits TLR7 activation^78^. The level of tRNA modification is altered during T-cell activation. It has been found that yW and ms^2^t^6^A modifications are significantly reduced during T cell proliferation, which may be to reduce the occurrence of ribosomal frameshifting and improve translational fidelity^79^. A recent study revealed that TRMT61A-mediated m^1^A modification of the tRNA 58 promotes the translation of multiple key proteins such as MYC by modulating codon decoding, to ensure a rapid immune response in CD4^+^ T cells (Figure 3B). This study, which for the first-time links tRNA-m^1^A_58_ modifications to functional changes in T cells, will provide new RNA epigenetic strategies for improving CD4^+^ T cell-mediated inflammatory responses and enhancing tumor immunotherapy efficacy^80^. T cell proliferation and activation involve complex molecular mechanisms, and the relationship between T cell homeostasis and tRNA modification is still largely unknown. It will be very interesting to explore how tRNA expression regulates immune response in the future, including not only tRNA modification and immune response, but also the relationship between the level of some tRFs and immune response, which would be a very valuable area of research for human health^8^, ^81^, ^82^.

### tRNA modifications and Diseases

The pathological consequences resulting from defects in tRNA modifications, known as 'RNA modopathies', occur in many tissues and cells^83^. Abnormal tRNA modifications affect normal translation by destabilizing tRNA, causing various diseases, including cancer, neurological diseases, diabetes, mitochondrial diseases, and so forth (Table 1). Studying the molecular mechanisms behind diseases is an essential process for diagnosing and treating diseases, but the complexity of genes requires us to make great efforts to keep exploring.

## Cancer

There is increasing evidence that some tRNA modifications and the expression of the enzymes they modify are associated with cancer progression. Here we mainly introduce the relationship between several common types of tRNA modifications and cancer, including m^7^G, m^6^A, m^1^A, mcm^5^U_34_, m^5^C, and Ψ. Since there is little evidence that other types of cancer-related RNA modifications are associated with tRNAs, we will not summarize them here.

### m^7^G in cancer

m^7^G located at position 46 is one of the most common tRNA modifications and has been found in eukaryotes, prokaryotes, and some archaea. Several recent studies have shown that the widespread m^7^G tRNA methylomes in mammals are related to the carcinogenesis and development of tumors^84^-^86^. The loss of METTL1 results in decreased m^7^G tRNA methylation and expression, inhibition of cell cycle and global translation, as well as the growth of tumors cell in many types, such as melanoma, liposarcoma (LPS), glioblastoma multiforme (GBM), and acute myeloid leukemia (AML). METTL1 mediates altered m^7^G-modified tRNAs and makes specific tRNAs enriched, especially tRNA^Arg^-TCT-4-1, which increases the translation of mRNAs with the AGA codon, thus regulating the cell cycle^87^, ^88^.

METTL1/WDR4 mediated m^7^G tRNA modification levels are increased in Intrahepatic cholangiocarcinoma (ICC) and are linked to poor prognosis, this may be because that METTL1/WDR4-mediated m^7^G tRNA modification in a selective manner affect the translation of oncogenic mRNAs, which have a higher frequency of m^7^G-related codons in ICC, such as genes involved in cell cycle and EGFR signaling pathway^58^. In the same way, the levels of METTL1/WDR4 are increased in hepatocellular carcinoma (HCC) and related to advanced clinical TNM stages and poor overall survival, indicating METTL1/WDR4 mediated m^7^G tRNA modification enhances the translation of target mRNAs through a codon-frequency-dependent mechanism^59^. A recent study linked m^7^G tRNA modification to radiotherapy resistance in HCC revealed the high level of METTL1 in tumor tissue is significantly related to poor prognosis in radiotherapy-treated patients with HCC^89^. Insufficient radiofrequency ablation is associated with a high recurrence of HCC. It has shown that m^7^G tRNA modification promotes the translation of SLUG/SNAIL under sublethal heat stress, and further makes the malignancy of METTL1 knockout HCC cells restored after sublethal heat exposure. This research elucidates that the METTL1-m^7^G-SLUG/SNAIL axis has the potential to be a therapeutic target for preventing metastasis of HCC after radiofrequency thermal ablation^90^. In addition, a recent study demonstrated that m^7^G tRNA modification is critical for enhancing lenvatinib resistance *in vivo*^91^, and METTL1 is implicated in 5-FU sensitivity in HeLa cells^92^. These studies suggest that m^7^G modification is associated with drug resistance of tumor cells, and provide a promising diagnostic marker and therapeutic target for drug resistance.

Not only in digestive tumors, but the METTL1 mediated m^7^G tRNA was also shown to enhance the development and malignancy of head and neck squamous cell carcinoma (HNSCC) via adjusting global mRNA translation of the PI3K/AKT/mTOR molecular axis, and shown to alter immune landscape^93^. Xiaoling Ying et al showed the pathological significance of METTL1 which is an oncogene in the development of bladder cancer (BC) through the METTL1-m^7^G-EGFR/EFEMP1 pathway^94^. In lung cancer, METTL1 mediated m^7^G tRNA modification and m^7^G tRNA decoded codon usage, enhances the translation of mRNA and in turn promotes lung cancer progression^95^. Further study found that METTL1 may be involved in the autophagy of A549 cells, and may act via the AKT/mTORC1 pathway in LUAD cells^96^. In nasopharyngeal carcinoma (NPC), METTL1/WDR4 and m^7^G tRNA modification enhance the EMT process of NPC cells through the WNT/β-catenin pathway to promote NPC progression, and METTL1 is linked to the chemosensitivity of NPC cells to cisplatin and docetaxel^97^. A recent study revealed METTL1-mediated tRNA m^7^G modification was an independent risk factor for neuroblastoma (NBL) patients and regulated mRNA translation of MTDH and PDCD10 in the form of m^7^G -related codon dependent manner to promote NBL progression in mechanism^98^. In esophageal squamous cell carcinoma (ESCC), a finding provides a novel translational regulatory mechanism mediated by m^7^G tRNA modification, which linked autophagy with translational machinery^99^. It is confirmed that METTL1 overexpression in the ESCC cells is closely related to RPTOR/ULK1 axis, and it could be an effective strategy to treat METTL1 overactive ESCC by targeting mTOR/ULK1/autophagy signaling pathway^100^. Taken together, these data demonstrated that m^7^G tRNA modification can impact the carcinogenesis and development of cancer in a variety of ways, METTL1 can be used as a marker for diagnosis and prognosis and therapeutic target in cancer.

### m^6^A in cancer

tRNA m^6^A methylation occurs at the nitrogen-6 position of adenine and is another important post-transcriptional modification^101^. This methylation is a dynamically reversible tRNA modification and can be reversed by demethylases, such as ALKBH5^102^, which promotes the development of GBM by enhancing the proliferation and self-renewal of tumor stem cells^103^. It is reported that tRNA modified by ALKBH3 demethylase improves protein translation efficiency in cells, which is essential for tumor proliferation^104^ and has been proposed as a possible therapeutic target for human pancreatic cancer^13^. But the m^6^A demethylase activity of ALKBH3 remains uncertain^104^, ^105^. In colorectal cancer, mutations of m^6^A regulators affect patient prognosis and may be associated with immune cell infiltration in colon tissues^106^. Besides, m^6^A RNA methylation regulators, as prognostic factors for colon and prostate cancer, have potential value for the treatment of related tumors and show high prospects in clinical cancer prognostic models^107^, ^108^. Of note, some studies of m^6^A in cancer require further exploration of whether they are tRNA modifications.

### m^1^A in cancer

m^1^A modification has been implicated in many cancers. It can enhance the migration, proliferation, and colony formation in cervical cancer and ovarian cancer cells^109^. The high-level expression of tRNA modifying enzyme hTrm6p/hTrm61p in BC tissue causes a large release of m^1^A in the urine, further promotes the proliferation of cells and inhibits cell apoptosis by influencing the modification of tRNA^Met^ in BC^110^, which consistent with findings in highly aggressive GBM^111^. The m^1^A on tRNA can be demethylated by ALKBH3, which promotes ribosome assembly and prevent apoptosis via regulating the biogenesis of tsRNA in HeLa, DU145, and PC3 cells^105^. Notably, m^1^A has been found to regulate tumor development through different pathways in non-small cell lung cancer, pancreatic cancer, prostate cancer, and liver cancer^112^-^115^, however, whether it is related to mRNA modifications or tRNA modifications needs further exploration.

### mcm^5^U_34_ /mcm^5^s^2^U_34_ in cancer

The 5-carbonylmethyluridine (cm^5^U) was methylated by human TRM9L and ALKBH8 to generate 5-methoxycarbonylmethyluridine (mcm^5^U) at U34 of tRNA. According to the report, the level of ALKBH8 expressed in BC is high, and ALKBH8 knockout promotes apoptosis by decreasing the protein expression of anti-apoptotic factors^116^. Another study reported that human TRM9L encodes a negative regulator of tumor growth that is often suppressed in a variety of cancers, including testicular, cervical, colorectal, bladder, breast, lung, and ovarian cancer^117^. In colon cancer, phosphorylated TRM9L links oxidative stress and cell cycle control and proliferation by interacting with 14-3-3 proteins, revealing TRM9L is a crucial downstream effector of the ERK molecular axis^117^. In ovarian cancer, TRM9L regulates the expression of LIN9 to activate pRB and p53 signaling pathways and thus inhibits the proliferation of ovarian cancer cells^118^. ELP3 and CTU1/2, which are tRNA modifying enzymes acting at position 34, are essential for the invasiveness and metastases of breast cancer and linked the tRNA-dependent translation of an ITAF to the IRES-dependent translation of a key oncogenic protein^119^. In addition, U_34_ tRNA modification is associated with drug resistance in melanoma. BRAF^V600E^ -expressing melanoma cells rely on U_34_ tRNA modifying enzymes for survival, and activation of tRNA modifying enzymes at position 34 promote the synthesis of HIF1α protein, resulting in enhanced glycolysis and conferring mTORC2‐dependent resistance to targeted BRAF inhibition. Thus, high levels of U_34_ tRNA modifying enzymes and HIF1α are related to the acquired resistance to anti-BRAF therapy^120^, ^121^.

### m^5^C in cancer

Similar to m^1^A, m^5^C is a powerful mechanism for regulating physiological processes in post-transcriptionally^122^. Dysregulation of m^5^C expression levels is common in various human cancers, but most current studies focus on mRNA, lncRNA or still unknown^123^, while m^5^C is the most abundant in tRNA and rRNA^124^. Studies have shown that m^5^C tRNA methyltransferases are associated with drug resistance of cancer cells. Co-overexpression of NSUN2 and METTL1 can enhance the cancer cell resistance to 5-FU by stabilizing tRNA and preventing rapid tRNA degradation (RTD) pathways^92^. The study found that the tRNA methyltransferase NSUN2 in HNSCC was significantly upregulated and increased the risk of death in HNSCC patients, indicating that NSUN2 is a potential therapeutic target and prognostic marker in HNSCC^125^. In addition, the interaction between NSUN2 expression and T cell activation status affects patient survival in HNSCC, suggesting that NSUN2 is a potential immune-related marker or therapeutic target, but the molecular mechanism behind it still needs to be further explored^126^. In ovarian cancer, NSUN2/IGF-II is associated with patient survival, and the subgroup of NSUN2^high^IGF-II^low^ has superior survival, whereas NSUN2^low^IGF-II^high^ has the worst^127^. Recently, Michaela Frye's group found that m^5^C and f^5^C modifications at mitochondrial tRNA^Met^ are required for the dynamic regulation of translation rate, and regulate the metabolic reprogramming of tumor cells, which is required for tumor invasion and metastasis from the primary site^128^.

### Ψ in cancer

Ψ is the most common epitranscriptomic modification. However, the biological role of Ψ that occurs on tRNA is largely unknown, especially in cancer. Studies have found PUS7 is highly expressed in GBM and predicts poor survival, moreover, PUS7 inhibitors can efficiently suppress GSC-derived tumor progression. Mechanistically, PUS7 regulates the codon-specific translational in GSCs by tRNA pseudouridylation, and regulates GSC growth via downregulating the TYK2-STAT1 pathway^129^. Currently, the inhibitor for pus7 has been identified to reduce Ψ levels of tRNA and inhibit GBM tumorigenesis and growth, providing potential therapeutic drugs for targeting PUS7 in GBM and other cancers^130^.

**Figure 4:**
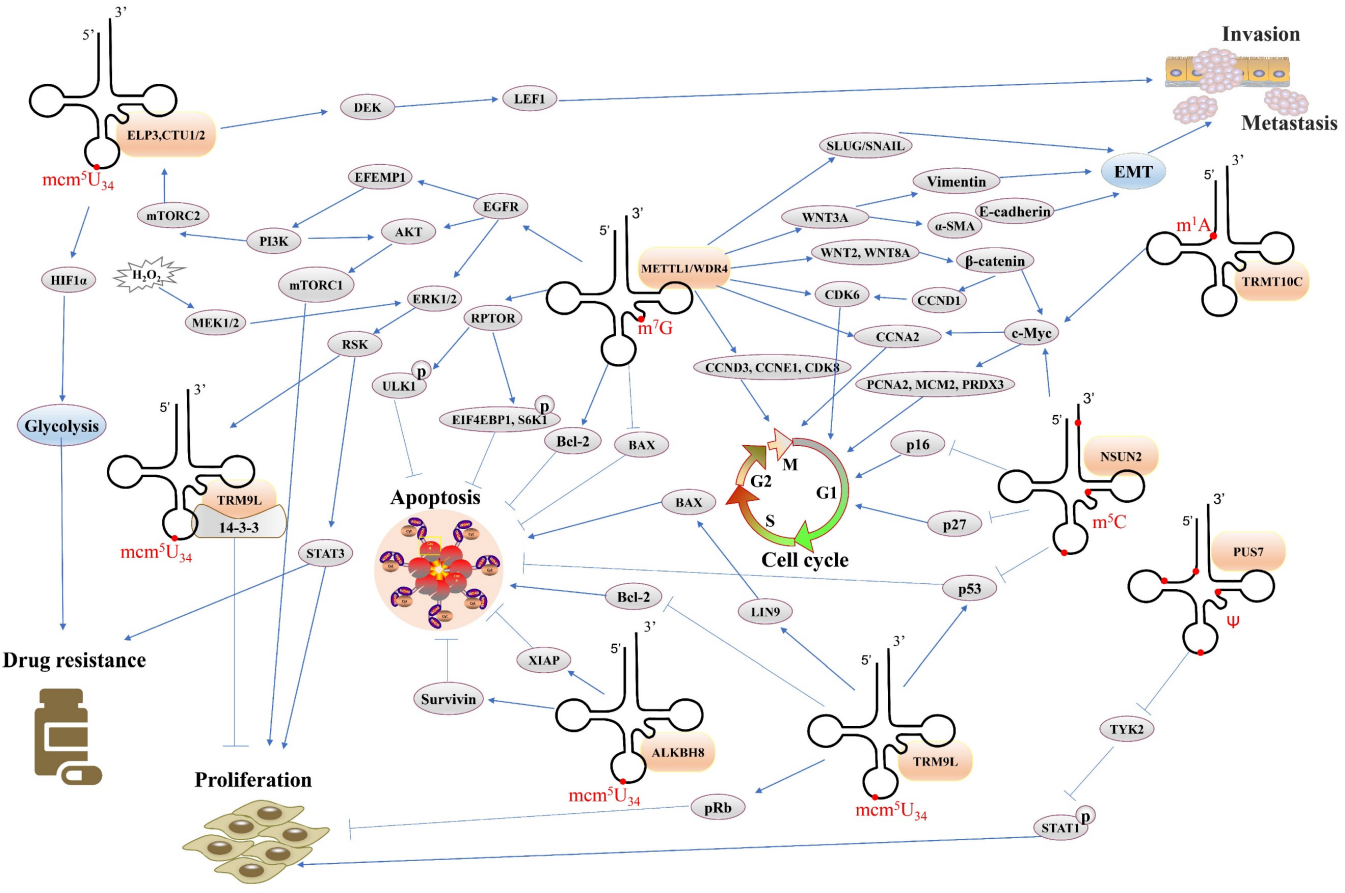
** Overview of the regulatory network of tRNA modifications in cancer. Notes:** Orange frames represent tRNA modifying enzymes. Gray frames represent protein molecules. EMT: epithelial-to-mesenchymal transition.

Overall, the study of tRNA modification is a new frontier in cancer research, which not only complements new content in cancer epigenetic regulation, provides new insights into tumor development, and molecular mechanisms of drug resistance, but also it promotes the development of new tumor treatments and may be ideal targets for cancer therapy. In this chapter, we summarize the function of common tRNA modifications in cancer (Table 2) and discuss the relevant regulatory mechanisms (Figure 4), which will contribute to the further study of cancer in the future.

## Neurological diseases

The effect of tRNA modifications on the nervous system is an emerging aspect of neuroscience. More and more genes involved in tRNA modifications have been proved to be related to neurodevelopmental disorders^131^, suggesting the increasing importance of tRNA modifications in human neural development. It is essential for us to know the functions of these tRNA modifying genes or enzymes, which will help us to realize their exact biological roles, and to develop new therapeutic strategies by controlling the expression of tRNA modifications related genes.

### Methylation modification and neurological diseases

In patients with mutations in the NSUN2 gene, the cells lacked the corresponding protein, resulting in the deficiency of site-specific 5-cytosine methylation at C47 and C48 of the tRNA^Asp^, and the patients present with moderate to severe intellectual impairment, facial deformities, and distal myopathy^124^, ^132^, ^133^. NSUN2-mediated tRNA methylation is indispensable for the migration and differentiation of intermediate progenitors in the ventricular zone toward the upper-layer neurons. In neural development, deficiency of NSUN2-mediated methylation increases the sensitivity of tRNA to ANG, resulting in the accumulation of more tRF-5 in the brain, which will interfere with differentiation of neuroepithelial stem cells, and reduce response sensitivity to growth factors and decrease the number of upper neurons, eventually resulting in neurodevelopmental defects^134^. Loss of NSUN2-mediated tRNA methylation causes a selective enrichment of tRF-5 in mice, impairs survival of striatal, cortical and hippocampal neurons by triggering cellular stress responses and cell death^71^. Recently, NSUN2-mediated tRNA cytosine methylation was found to be related to emotion. NSUN2 overexpression in the cerebral cortex produces depression-like behavior, and NSUN2 deletion produces an anti-depressant phenotype^135^. Patients with NSUN3 mutations exhibit early-onset mitochondrial encephalopathy and seizures, possibly due to impaired mt-tRNA^Met^ methylation affecting mitochondrial translation^136^, ^137^.

Recent studies have shown heterotropic m^1^A methylation in the mitochondrial tRNA of these mice by mapping the abundant m^1^A tRNA modification in the cerebral cortex of mouse models of Alzheimer's disease^138^. Silzer TK et al also reported significant hypermethylation of specific sites at position 9 of mt-tRNA in the cerebellar tissue of individuals with progressive supranuclear palsy and Alzheimer's disease^139^, suggesting that hypo m^1^A modification of mt-tRNA is linked to neurodegenerative diseases.

In addition, the lack of DARLD3 in human cells blocks the m^3^C formation of tRNA^Arg^. It will suffer from early-onset epileptic encephalopathy and severe developmental delay if human individuals with a deficiency of the DALRD3 gene, indicating that m^3^C-mediated tRNA modification in mammalian tRNA^Arg^ is related to nervous system function^140^.

Studies have shown that m^7^G tRNA modification is involved in neural differentiation and brain development. Mutations in WDR4, which interacts with METTL1 to form a complex, result in defective m^7^G-mediated tRNA modification and interfere with the correct expression of genes on the neural spectrum^57^. Biallelic variants occurring in the WDR4 gene lead to microcephalic primordial dwarfisms, characterized by severe growth retardation and microcephaly^141^.

The latest case report shows biallelic variant in the domain of ALKBH8 causes syndromic intellectual disability, adding a new variant to the ever-expanding list of tRNA modifications related to ALKBH8^142^.

### ac^4^C and the nervous system

THUMPD1 is participated in regulating ac^4^C modification of tRNA. In a recent study, Martin Broly et al reported that 13 individuals from 8 families had a rare mutation of THUMPD1 dysfunction that resulted in a deficiency of ac^4^C modification of individually purified tRNA^Ser^-CGA^143^. The results of research show that the loss of tRNA acetylation due to lack of THUMPD1 function leads to syndromic intellectual disability, this is because the lack of tRNA modification may lead to impaired protein synthesis and thereby affecting the proteostasis at important stages of neural development.

### Ψ and the nervous system

PUS3 mutations can cause a rare neurodevelopmental disorder^144^. Recently, a novel homozygous truncating mutation in *PUS3* was found to be associated with intellectual impairment, patients with this mutation had dropped levels of uracil isomerization at tRNA positions 38 and 39^145^. Patients with the PUS7 mutation showed delayed speech and aggressive behavior, and experiments in fruit flies demonstrated that this behavioral deficit was caused by the PUS7 mutation^146^.

Furthermore, the adenosine deaminase tRNA-specific 2/3 (ADAT2/ADAT3) complex can catalyze adenosine deamination, called inosine. It can lead to an autosomal recessive genetic mental disorder if ADAT2/3 is mutated^147^, ^148^. The EPL3, a subunit of the elongator complex that modifies the uridine at the tRNA wobble position, is low expressed in the motor cortex of amyotrophic lateral sclerosis (ALS), which may be related to mcm^5^s^2^U-modified tRNA levels^149^.

## Type 2 diabetes

The study of diabetes genetics has changed due to rapid advances in sequencing technologies. Among the risk factors associated with diabetes, some disorders of tRNA modifications are thought to be related to type 2 diabetes^27^.

CDKAL1, a tRNA methylthiotransferase, is located at position 37 of cytoplasmic tRNA^Lys^^150^, ^151^. Genetic variations in the CDKAL1 gene are linked to type 2 diabetes in different ethnic groups. It showed hypertrophy of islets, reduced insulin secretion, and impaired glycemic control, which are typical phenotypes related to type 2 diabetes in CDKAL1 knockout mice^150^. Misreading of the Lys codon in CDKAL1-deficient β cells leads to reduced glucose-stimulated proinsulin synthesis, this may be related to the 2-methylthio-modification (ms^2^-) of N^6^-threonylcarbonyladenosine at position-37 (ms^2^t^6^A_37_) in tRNA_UUU_^Lys3^^152^. Insulin levels *in vivo* are regulated by ferrimodulin 2 (Irp2) through iron-mediated CDKAL1-catalyzed tRNA modification. The loss of Irp2 leads to impaired iron-sulfur cluster biosynthesis and functional iron deficiency in the β-cells, and reduces the activity of ferrithionein CDKAL1, resulting in decreased insulin synthesis^153^, ^154^. These findings suggest that a fully modified tRNA^Lys^ (UUU) may be required for the correct translation of insulin mRNA.

Interestingly, TRMT10A, a tRNA methyltransferase, methylates guanosine at position 9 of tRNA, has been proved that lack of TRMT10A sensitizes β-cells to apoptosis^155^. Recently, several cases have been reported that TRMT10A is associated with childhood diabetes. In addition to diabetes, the patients also suffered from microcephaly and intellectual disability, and genotype testing suggested homozygous mutations in the TRMT10A gene^156^-^158^.

## Mitochondrial disease

Mitochondrial disease, also known as mitochondrial encephalopathy, is a disorder caused by mitochondrial dysfunction with diverse clinical phenotypes, including blindness, deafness, dyskinesia and myopathy^159^, ^160^. The absence of tRNA modification in mitochondria often leads to pathological consequences^161^. For example, a missense mutation in PUS1 is associated with the disorder mitochondrial myopathy with lactic acidosis and sideroblastic anemia (MLASA) in humans^162^. Besides, mutations in TRMT10C affect mt-tRNA processing and mitochondrial protein synthesis, leading to mitochondrial diseases including deafness in newborns^163^.

NSUN3 is an m^5^C methyltransferase of mt-tRNA, specifically for the 'wobble' 34th position of mt-tRNA^Met^^137^. Most mt-tRNA^Met^-C34 forms m^5^C and further oxidized by ALKBH1 to generate f^5^C^164^, ^165^. Deficiency of NSUN3 or ALKBH1 affects mitochondrial translation, results in reduced cell proliferation and may be linked to early-onset mitochondrial encephalomyopathy and seizures^136^.

Mitochondrial myopathy, encephalopathy, lactic acidosis, and stroke-like episodes (MELAS) and Myoclonic Epilepsy with Ragged Red Fibers (MERRF)^166^ are a group of mitochondrial diseases caused by the lack of taurine modification at the first anticodon nucleotide of mt-tRNA^Leu(UUR)^ 167. Due to the absence of naturally occurring modified nucleoside 5-taurinomethyluridine (τm^5^U) located at the wobble position (C34) of tRNA^Leu^, resulting in errors in codon translation^168^. High dose of taurine can improve mitochondrial tRNA^Leu (UUR)^ modification defect in peripheral blood leukocytes and inhibit stroke in MELAS patients^169^. Mtu1(Trmu) is a kind of highly conserved tRNA modifying enzyme, and associated with the modification of τm^5^s^2^U at the 'wobble' 34th position of tRNA^Lys^, tRNA^Glu^ and tRNA^Gln^^170^. These abnormal modifications affect the translation function by affecting mitochondrial respiration and thus lead to auditory defects in zebrafish^170^ and suppressed osteogenic differentiation in mice^171^.

## Conclusion & perspective

In summary, the researches on the biological functions of tRNA modifications have made great progress, and become a hot topic in the field of RNA modifications. But there are still many problems to be explored.

The detection technologies of tRNA modifications have limited the development of research. Due to the rich variety of tRNA modification types and their elusive function, the detection, quantification and localization of these tRNA modifications are crucial for understanding the elusive function of tRNA modifications. For the traditional high-throughput RNA sequencing technology, on the one hand, they will affect the preparation of the RNA-seq library, and on the other hand, they cannot implement deep sequencing for some highly modified tsRNA. To solve the problems of existing sequencing technologies, new technologies have been developed and applied, such as PANDORA-Seq^172^, mim-tRNA seq^173^ and Absolute Quantification RNA-seq^174^. These new techniques alleviate the bias in traditional tRNA sequencing, accurately measure the abundance of tRNA in cells, and reveal the quantitative map of tRNA modifications. However, there are still some unavoidable limitations, and there is no general and precise method to quantify the modification in tRNA. We urgently need to develop more sensitive and reliable detection methods to rapidly and quantitatively detect tRNA modifications, and open new avenues for exploration of more types of RNA modifications in the future.

tRNA modification is driven by various modifying enzymes, and a variety of modifying enzymes directly affect the biological function of tRNA. The existence of tRNA modification enhances the stability of tRNA structure, improves the accuracy of decoding and the efficiency of translation during protein synthesis, and plays an important role in cell cycle and stress. But our understanding of how tRNA modifications facilitate these processes remains incomplete. Considering that the spatiotemporal order of tRNA modifications plays an integral role in its function^175^, ^176^, but related studies are still very rare, it would be interesting to investigate the synergistic mechanism of different modifying enzymes. The tRNA acetylation has also been reported to play an essential role in antibiotic resistance^177^, and other types of tRNA modifications were mentioned above for chemoresistance in tumor patients. Therefore, the mechanism of tRNA modifications in drug resistance and immune escape is also worthy of further exploration. In recent years, with the deepening of related studies on tRNA modifications, its effects on human diseases are gradually being revealed^22^, especially the effect of tRNA modifying enzymes on cancer. Although there is evidence that aberrant expression of some modifying enzymes is significantly associated with human pathological processes^178^, the specific molecular pathways and interactions between tRNA modifications and other noncoding RNA modifications should be further explored. All in all, these studies will help us understand the function of tRNA modifications in epigenetics.

What is more, the clinical potential of tRNA modifying enzymes has also attracted widespread attention. Studies have revealed that levels of tRNA modification are related to tumor progression, and some high levels of tRNA modifications can be detected in the urine or blood of cancer patients, suggesting that tRNA-modifying enzymes may be new markers for cancer diagnosis and prognosis (Table 3). In addition, tRNA-modifying enzymes and their inhibitors can promote or suppress tumor cell phenotypes, implying their potential as new therapeutic targets. With the development of precision medicine, it will be an attractive strategy for human cancer treatment that targeting tRNA modification systems. However, this kind of research is still in its infancy, what we know about the basic biological functions of tRNA modifications still has a large gap, and there are still limitations in the development of tRNA modifying enzyme inhibitors. We still have a long way to go before it can be applied to clinical practice.

Our ultimate goal is to solve human pain, so it is the top priority to explore the mechanism of action of various modifications and modified enzymes in various diseases, so as to develop targeted drugs to provide new possibilities for disease treatment or to provide new markers for disease diagnosis. In conclusion, we need to reveal the occurrence and function of tRNA modifications from different perspectives.

## Figures and Tables

**Table 1:** Summary of tRNA modifications in human diseases

tRNA modifications	Diseases	References
m^7^G	GBM, LPS, Melanoma, AML, ICC, HCC, Cervical cancer, HNSCC, BC, LC, Microcephalic primordial dwarfisms, NPC, NBL, ESCC	58, 59, 87-89, 92-95, 97-99
m^6^A	GBM, Pancreatic cancer	103
m^1^A	Cervical cancer, Prostate cancer, HCC, BC, GBM, Alzheimer's disease, Mitochondrial disorders	105, 109-111, 138, 163
mcm^5^U_34_/mcm^5^s^2^U_34_	BC, Breast cancer, Ovarian cancer, Colon cancer, Melanoma	116-118, 120
m^5^C	HNSCC, Ovarian cancer, ID, depression-like behavior, Dubowitz-like syndrome	124, 125, 127, 132, 135
m^3^C	Developmental delay and early-onset epileptic encephalopathy	140
ac^4^C	Syndromic intellectual disability	143
PUS	GBM, Neurodevelopmental disorder, Mental disorder, ALS, MLASA	129, 145, 147, 149, 162
m^1^G	Childhood diabetes	156
ms^2^t^6^A_37_	Type 2 diabetes	152
τm^5^U	MELAS	167

**Abbreviations:** GBM: glioblastoma multiforme; LPS: liposarcoma; AML: acute myeloid leukemia; HCC: hepatocellular carcinoma; ICC: intrahepatic cholangiocarcinoma; HNSCC: head and neck squamous cell carcinoma; NPC: nasopharyngeal carcinoma; ESCC: esophageal squamous cell carcinoma. LC: Lung cancer; BC: bladder cancer; NBL: neuroblastoma. ID: Intellectual Disabilities; ALS: amyotrophic lateral sclerosis; MLASA: mitochondrial myopathy with lactic acidosis and sideroblastic anemia; MELAS: mitochondrial encephalomyopathy, lactic acidosis, and stroke-like episodes.

**Table 2:** Function of tRNA modifications in cancer

tRNA modifications	Cancers	tRNA modifying enzymes	Molecular axis	Function	References
m^7^G	GBM, Melanoma, Liposarcoma, AML	METTL1 (↑)	Cell cycle related proteins	Change the cell cycle, promote tumor growth	87, 88
	HCC	METTL1/WDR4 (↑)	EGFR, CCNA2	Promote cell proliferation, migration and invasion	59
	HCC	METTL1/WDR4 (↑)	SLUG/SNAIL	Promote HCC metastasis under sublethal heat exposure	90
	HCC	METTL1/WDR4 (↑)	EGFR-ERK-STAT3	Promote cell proliferation and inhibit apoptosis	91
	ICC	METTL1/WDR4 (↑)	EGFR, AKT-mTOR, Cell cycle related proteins	Promote cell growth and colony formation, inhibit apoptosis, promote migration and invasion	58
	HNSCC	METTL1/WDR4 (↑)	PI3K-AKT-mTOR	Promote cell proliferation, migration and invasion, promote cell colony formation, inhibit apoptosis	93
	Bladder cancer	METTL1 (↑)	EGFR/EFEMP1	Promote cell proliferation, migration and invasion	94
	Lung cancer	METTL1 (↑)	AKT/mTORC1	Promote cell proliferation and colony formation, promote migration and invasion	95, 96
	NPC	METTL1 (↑)	ANRT/METTL1/WNT/EMT	Promote cell proliferation and colony formation, inhibit apoptosis, promote migration and invasion, and promote drug resistance	97
	Neuroblastoma	METTL1 (↑)	MTDH, PDCD10	Promote cell proliferation and inhibit apoptosis	98
	ESCC	METTL1 (↑)	RPTOR/ULK1	Promote cell proliferation and colony formation, inhibit apoptosis	99, 100
m^6^A	Pancreatic cancer	ALKBH3 (↑)	Global translation	Promote cell growth	104
	Cervical cancer	TRMT10C (↑)	c-Myc related pathway	Promote cell proliferation, migration and colony formation	109
m^1^A	Cervical cancer, Prostate cancer, HCC	ALKBH3 (↑)	Enhance the sensitivity of tRNA to ANG and promote the binding of tDR and Cyt-c	Promote cell proliferation and inhibit apoptosis	105
	GBM	hTRM6p/61p (↑)	PKCα related pathway	Promote cell colony formation and pelleting ability, and promote invasion	111
mcm5U34 /mcm5s2U34	Bladder cancer	ALKBH8 (↑)	Survivin/XIAP	Promote cell proliferation and inhibit apoptosis	116
	Colon cancer	TRM9L (↓)	H_2_O_2_/ERK/RSK/14-3-3	Inhibit cell proliferation	117
	Ovarian cancer	TRM9L (↓)	LIN9/Bax/Bcl-2	Promote cell apoptosis and inhibit cell proliferation	118
	Breast cancer	ELP3, CTU1/2 (↑)	ELP3-CTU1/2-DEK-IRES-LEF1	Promote cell invasion and metastasis	119
	Melanoma	ELP1, ELP3, CTU1/2 (↑)	PI3K/mTORC2	Promote drug resistance	120, 121
m^5^C	HNSCC	NSUN2 (↑)	c-Myc related pathway	Promote tumor growth	125
Ψ	GBM	PUS7 (↑)	TYK2/STAT1	Promote cell growth and self-renewal	129, 130

Notes: ↑, upregulated; ↓, downregulated. **Abbreviations:** EMT: epithelial-mesenchymal transition; ANG: angiotensin; GBM: glioblastoma multiforme; AML: acute myeloid leukemia; HCC: hepatocellular carcinoma; ICC: intrahepatic cholangiocarcinoma; HNSCC: head and neck squamous cell carcinoma; NPC: nasopharyngeal carcinoma; ESCC: esophageal squamous cell carcinoma.

**Table 3:** Clinical potential of tRNA modifying enzymes in cancer

Modifying enzymes	Cancer	Clinical potential	References
METTL1	HCC	Promote radiotherapy resistance of HCC and is significantly associated with poor prognosis of HCC after radiotherapy	89
METTL1	HCC	Promote lenvatinib resistance in HCC	91
METTL1/WDR4	HCC	Overexpressed in HCC, correlated with tumor stage and patient survival	59
METTL1/WDR4	HCC	Insufficient radiofrequency ablation promotes HCC metastasis	90
METTL1/WDR4	ICC	Overexpressed in ICC, and patients with high expression have poor survival and are more likely to relapse	58
NSUN2, METTL1	Cervical cancer	Combined knockdown of NSUN2 and METTL1 significantly enhanced the sensitivity of Hela cells to 5-FU	92
METTL1/WDR4	HNSCC	Overexpressed in HNSCC and correlates with tumor stage and overall survival	93
NSUN2	HNSCC	Overexpressed in HNSCC, leads to short overall survival and a high risk of death	125
NSUN2	HNSCC	Interaction with T cell activation, affects the survival of HNSCC patients	126
NSUN2	Ovarian cancer	Overexpressed in ovarian cancer, and it has the worst survival in the low NSUN2 and high IGF-II subgroups	127
METTL1	BC	Overexpressed in BC, and patients with high expression have poor disease-free survival	94
hTRM6p/61p	BC	Promote the high discharge of m^1^A in the urine of BC patients	110
ALKBH8	BC	Overexpressed in advanced bladder cancer	116
METTL1/WDR4	LUAD	Overexpressed in LUAD and leads to poor survival	95, 96
METTL1	NPC	Overexpressed in NPC, which is related to TNM stage and patient survival, and promotes drug resistance	97
METTL1	NBL	Overexpressed in advanced NBL and leads to poor prognosis	98
METTL1	ESCC	Overexpressed in ESCC, correlates with patient survival and tumor TNM stage	99
ALKBH3	Pancreatic cancer	Overexpressed in pancreatic cancer and knockdown inhibits tumor cell growth	104
ELP1, ELP3, CTU1/2	Melanoma	Promote drug resistance in melanoma	120, 121
PUS7	GBM	Overexpressed in GBM and leads to poor survival	129, 130

**Abbreviations:** GBM: glioblastoma multiforme; HCC: hepatocellular carcinoma; ICC: intrahepatic cholangiocarcinoma; HNSCC: head and neck squamous cell carcinoma; NPC: nasopharyngeal carcinoma; ESCC: esophageal squamous cell carcinoma. LUAD: Lung adenocarcinoma; BC: bladder cancer; NBL: neuroblastoma.

## Abbreviations

tRNA: transfer RNA
mRNA: messenger RNA
tsRNA: tRNA-derived small RNA fragments
tiRNA: tRNA-derived stress-induced RNA
tRF: tRNA-derived fragment
m^5^U: 5-Methyluridine
ac^4^C: N^4^-acetylcytidine
m^1^A: N1-methyladenosine
m^5^C: 5-methylcytodine
m^2^G: N2-methylguanosine
m^2^_2_G: N2, N2-dimethylguanosine
t^6^A: N6-Threonylcarbamoyladenosine
m^7^G: N7-methylguanosine
m^1^G: N1-methylguanosine
m^3^C: 3-methylcytosine
mcm^5^U: 5-methoxycarbonylmethyluridine
mcm^5^s^2^U: 5-methoxycarbonylmethyl-2-thiouridine
Q_34_: Queuosine 34
m^5^U: 5-methyluridine
acp^3^U_47_: 3-(3-amino-3-carboxypropyl) uridine U_47_
f^5^C: 5-formylcytidine
m^6^A: N6-methyladenosine
ms^2^t^6^A: 2-methylthio-modification of N^6^-threonylcarbonyladenosine
Gm: Guanosine 2′-O-methylation
DNMT2: DNA methyltransferase2
TRM10: transfer RNA methyltransferase 10
NSUN: NOP2/Sun RNA methyltransferase
METTL1: methyltransferase like 1
WDR4: WD Repeat Domain 4
THUMPD1: THUMP Domain Containing 1
PUS: Pseudouridine Synthase
TRM9L: transfer RNA methyltransferase 9-like gene
DARLD3: domain containing 3
ALKBH: ALKB homolog
THUMPD1: THUMP Domain Containing 1
CDKAL1: CDK5 Regulatory Subunit Associated Protein 1 Like 1
TRMT10A: TRNA Methyltransferase 10 Homolog A
TRMT61A: TRNA Methyltransferase 61 Homolog A
TRMU: tRNA mitochondrial 2-thiouridylase
TRMT2A: TRNA Methyltransferase 2 Homolog A
ROS: reactive oxygen species
MELAS: mitochondrial encephalomyopathy, lactic acidosis, and stroke-like episodes
ESCs: embryonic stem cells
ICC: intrahepatic cholangiocarcinoma
GBM: glioblastoma multiforme
LPS: liposarcoma
AML: acute myeloid leukemia
HCC: hepatocellular carcinoma
HNSCC: head and neck squamous cell carcinoma
BC: bladder cancer
LC: lung cancer
NPC: nasopharyngeal carcinoma
ESCC: esophageal squamous cell carcinoma
ID: Intellectual Disabilities
ALS: amyotrophic lateral sclerosis
MLASA: mitochondrial myopathy with lactic acidosis and sideroblastic anemia
MERRF: Myoclonic Epilepsy with Ragged Red Fibers
NBL: neuroblastoma
LUAD: Lung adenocarcinoma
ANG: angiotensin
RTD: rapid tRNA degradation
EMT: epithelial-mesenchymal transition
